# DNA Sequence Variants in the Five Prime Untranslated Region of the Cyclooxygenase-2 Gene Are Commonly Found in Healthy Dogs and Gray Wolves

**DOI:** 10.1371/journal.pone.0133127

**Published:** 2015-08-05

**Authors:** Noa Safra, Louisa J. Hayward, Miriam Aguilar, Benjamin N. Sacks, Jodi L. Westropp, F. Charles Mohr, Cathryn S. Mellersh, Danika L. Bannasch

**Affiliations:** 1 Department of Population Health and Reproduction, School of Veterinary Medicine, University of California Davis, Davis, California, United States of America; 2 Animal Health Trust, Newmarket, United Kingdom; 3 Veterinary Genetics Laboratory, School of Veterinary Medicine, University of California Davis, Davis, California United States of America; 4 Department of Veterinary Medicine and Epidemiology, School of Veterinary Medicine, University of California Davis, Davis, California, United States of America; 5 Department of Pathology, Microbiology and Immunology, School of Veterinary Medicine, University of California Davis, Davis, California, United States of America; Faculty of Animal Sciences and Food Engineering, University of São Paulo, Pirassununga, SP, Brazil, BRAZIL

## Abstract

The aim of this study was to investigate the frequency of regional DNA variants upstream to the translation initiation site of the canine Cyclooxygenase-2 (*Cox-2*) gene in healthy dogs. *Cox-2* plays a role in various disease conditions such as acute and chronic inflammation, osteoarthritis and malignancy. A role for *Cox-2* DNA variants in genetic predisposition to canine renal dysplasia has been proposed and dog breeders have been encouraged to select against these DNA variants. We sequenced 272–422 bases in 152 dogs unaffected by renal dysplasia and found 19 different haplotypes including 11 genetic variants which had not been described previously. We genotyped 7 gray wolves to ascertain the wildtype variant and found that the wolves we analyzed had predominantly the second most common DNA variant found in dogs. Our results demonstrate an elevated level of regional polymorphism that appears to be a feature of healthy domesticated dogs.

## Introduction

Cyclooxygenase *(Cox)* is the key enzyme in the biosynthesis of prostaglandins, which play a significant role in health and disease in the kidney, gastrointestinal tract, and in the skeletal and ocular systems [1]. There are two isozymes of *Cox*: a constitutively active form, *Cox1* and an inducible form, *Cox2*. *Cox-2* expression is regulated by stimulating conditions such as inflammation, injury or pain and it is upregulated during disease stages [2]. *Cox-2* sequence variants that might modify the levels of protein expression would be expected to influence disease phenotype, and *Cox-2* has been tested for associations with over 60 disorders with more than 1000 articles citing this gene in PubMed [3].

A role for *Cox-2* DNA variants in canine renal dysplasia has been proposed since sequence variants were identified within the 5’ UTR region of canine *Cox-2* in affected dogs [4]. Canine renal dysplasia (RD) is a progressive heritable nephropathy affecting many dog breeds [5–18]. Clinically, RD is characterized by clinical signs associated with chronic renal failure [17, 19, 20]. The age of onset of clinical signs varies between 3 weeks to ~3 years of age [16], and disease severity ranges between individuals [21], making this disorder challenging to diagnose [19].

The gold standard for diagnosis relies on renal biopsy and histopathological examination of a surgical wedge or a necropsy sample. It has been suggested that in order to make a definitive diagnosis, the sample must include at least 100 glomeruli, and the complete architecture of the renal cortex has to be evaluated [18, 20, 21]. Histologically, renal dysplasia can demonstrate a range of structural changes and includes findings inappropriate to the developmental stage of the dog examined [22]. There are 5 main morphologic characteristics of canine renal dysplasia, and the presence of at least one of them is required in order to obtain the diagnosis. They include asynchronous differentiation of nephrons (immature “fetal” glomeruli and tubules); persistent mesenchyme; persistent metanephric ducts; atypical tubular epithelium; and dysontogenic metaplasia [21].

Obtaining a diagnosis of renal dysplasia without a renal biopsy is difficult. While ultrasonography has been suggested as a screening tool, the changes noted ultrasonographically are not pathognomonic for renal dysplasia and can be found with other renal disorders [23]. Detecting mild or sub-clinical cases becomes even more difficult for clients and for breeders wanting to screen their dogs. Consequently, sub-clinical cases go undetected and are used in breeding programs, which may explain the high frequency of RD within certain breeds like the Lhasa Apso and the Shih-Tzu [4]. The high incidence of disease within certain breeds is suggestive of a familial disease, and both an incompletely penetrant autosomal dominant mode and a recessive mode of inheritance have been proposed [24]. Based on the presence of DNA variants in the 5’UTR of *Cox-2* in RD affected dogs, a commercially available DNA test has been offered to dog breeders (http://www.dogenes.com/). However, the causative nature of these DNA variants has come into question [25].

In order to ascertain the frequency of sequence variants at the 5’UTR of canine *Cox-2*, we evaluated sequences from dogs free of renal disease, and from gray wolves; the dog’s direct wild ancestor [26, 27]. Our findings suggest that regional variants are a common finding in dogs and do not present a genetic risk for RD.

## Materials and Methods

DNA samples (n = 22) from dogs (Canis familiaris), obtained from patients of the Veterinary Medical Teaching Hospital at UC Davis that underwent complete necropsy and did not have histological evidence of RD were analyzed. Additional samples (n = 46) came from dogs 6 years old or older that had normal blood urea nitrogen (BUN) and creatinine concentrations (values were recorded during routine serum biochemical analysis). Samples of Flat-Coated Retrievers (FCRs) (n = 84) from the DNA collection of the Kennel Club Genetics Centre at the Animal Health Trust (AHT) in Newmarket, UK (submitted to the AHT with owner consent for use in variable research projects), were also used. The cohort of 84 FCRs ranged in age from 5 months to 14 years at the time of sample submission, and each dog had unique parentage (168 different dams and sires are represented). These dogs were free of renal disease at the time of sample submission, according to owners. Additional samples (n = 12) came from gray wolves (Canis lupus) collected from the Yukon Territory, Canada. These samples were archived for over ten years and used previously [28]; no samples were collected specifically for the purpose of this study and no animals were killed specifically for this study. The wolf samples were genotyped to ensure that our analysis was restricted to independent (i.e. unrelated) wolves. A panel of 24 short tandem repeat (STR) markers representing 20 canine autosomes was used by the Veterinary Genetics Laboratory (VGL) as was previously described [29]. The gray wolf relatedness was estimated using a maximum likelihood approach [30], and individuals with estimated relatedness <0.15 were considered to be unrelated which left 7 wolves for the analysis. Genomic DNA was extracted from EDTA whole blood or tissue samples using Gentra Puregene DNA purification extraction kit (Qiagen, Valencia, CA) or the Nucleon Genomic DNA Extraction Kit (Tepnel Life Sciences, Manchester, UK). Buccal swab DNA was extracted using the QIAamp DNA Blood Midi Kit (Qiagen, Valencia, CA).

The published primers [4]: 1-F: 5'-TTGTCAAACAACTTGCAGCGAGCG; 1-R: 5'-ATCACCCAGCCGAGGAGTC that amplify 272 bases were used to genotype the FCRs (n = 84), and 2-F: 5′ GCCCTTTACCAGGTCAAACA; 2-R: 5′ GCGAGGACCCAACAGCTTAC that amplify 422 bases were used to genotype the rest of the samples (n = 68). Reaction mixes using primers 1F and 1R consisted of 0.2 mM dNTPs, 1x PCR buffer, 1x Q solution (Qiagen), 0.8 μM of each primer, 0.5 U HotStarTaq Plus DNA Polymerase (Qiagen), 2 μl genomic DNA, and ultrapure water to give a final volume of 12 μl. Cycling conditions for PCR were 3 minutes at 95°C, followed by 35 cycles of 30 seconds at 95°C, 30 seconds at 60°C, and 1 minute at 72°C, with a final extension of 10 minutes at 72°C. Products were sequenced using BigDye Terminator v3.1 on an ABI 3130xl Genetic Analyzer (Applied Biosystems). Sequence traces were visually analyzed using the Staden Gap4 package [31]. Primers 2F and 2R (Tm = 68°C) were used to generate PCR products using AccuPrime GC-Rich DNA Polymerase (AccuPrime high GC DNA polymerase kit; life technologies, Grand Island, NY 14072, USA). PCR cycle conditions, Sanger sequencing protocol and sequence analysis were previously described [32]. For heterozygous individuals, PCR products were cloned, sequenced, and analyzed as was previously described [32].

## Results

We investigated the presence of DNA sequence variants within the 5’UTR of the canine *Cox-2* gene in FCRs reported free of renal disease by owners (n = 84) and in samples from dogs free of RD based on their clinical biochemistry or necropsy results (n = 68). Forty six dogs with serum BUN (reference range 6–31 mg/dL), creatinine (reference range 0.5–1.6 mg/dL) concentrations within the reference ranges, and no clinical signs associated with renal disease; and 22 dogs had necropsy results confirming absence of RD. The entire cohort consisted of 142 pure-bred dogs from 30 different breeds, and of 10 mixed-bred dogs (S1 Table).

**Fig 1:**
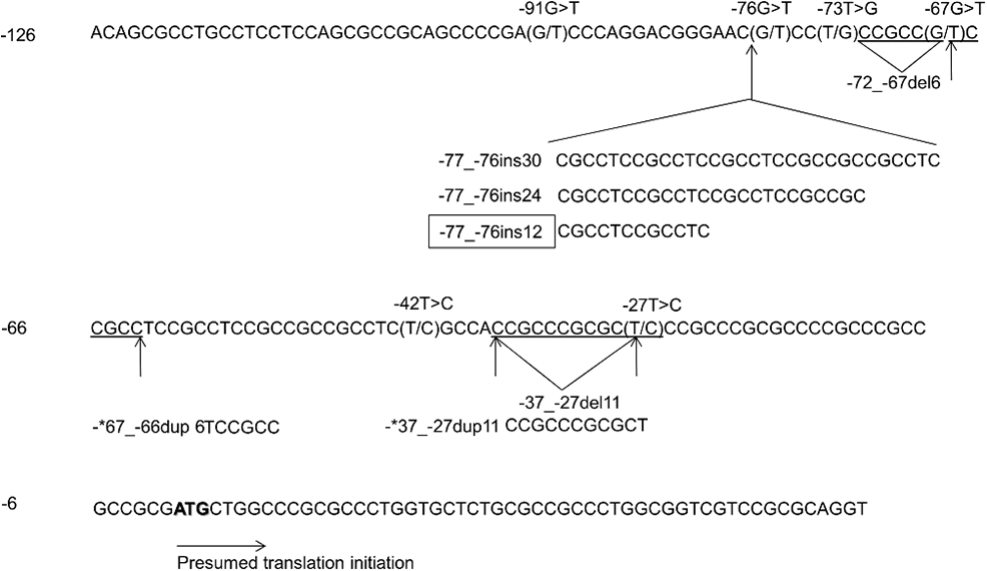
DNA sequence variants identified within the 5’UTR of the *Cox-2* gene in 152 dogs. Base positions upstream of the ATG translation initiation site (position zero) are noted on the left. *The exact base position for the duplicated sequence could not be determined. The sequence variant most commonly found in gray wolves is boxed.

The first coding ATG, according to dog mRNA sequence (GenBank: AY044905) represents position zero. The sequence variants identified upstream to position zero are described based on standard mutation nomenclature recommended by the Human Genome Variation Society (HGVS) [33], and in relation to CanFam3.1 sequence assembly. However, because of the repetitive high-GC content of the 5’UTR sequence of the *Cox-2* gene, the exact base positioning of certain sequence variants could not be determined (Fig 1, S1 Table).

Table 1 lists the variants found in the cohort of 68 dogs by nucleotide position, variant type and haplotype frequency. S2 and S3 Tables list the haplotypes observed within the subgroups of dogs without evidence of azotemia (n = 46), and dogs that were found free of RD by necropsy (n = 22). There were no statistically significant differences between the frequencies of the variants found within these two groups (Fisher exact test p value = 0.658842). The majority of the dogs (n = 44) had at least one haplotype identical to the CanFam3.1 assembly sequence, and the frequency of the CanFam3.1 haplotype was 0.4 in this sample set. Sequence variant *Cox2*–77_-76ins12 was the second most common at a frequency of 0.3. Recombined haplotypes and novel variants were commonly observed and a total of 17 haplotypes were observed in the 68 dogs. Listed in Table 1 are 11 genetic variants which had not previously been described.

**Table 1:** 5’UTR *Cox-2* variants found in 68 dogs free of renal disease^*^.

Haplotype	# of dogs	# of haplotypesʵ	Frequencyʞ
CanFam3.1 assembly sequence	44	55	0.4
-77_-76ins12	27	38	0.3
-72_-67del6; -42T>C; -37_-27del11	8	12	0.08
-77_-76ins24	4	6	0.04
*-77_-76ins30	4	6	0.04
-37_-27del11	3	3	0.02
-77_-76ins12; -77_-76ins24	1	2	0.014
-72_-67del6; -42T>C; *-37_-27dup11	1	2	0.014
-77_-76ins12; -42T>C; -37_-27del11	1	1	0.01
*-67G>T; -42T>C; -37_-27del11	1	1	0.01
-77_-76ins12; -72_-67del6; -37_-27del11	1	1	0.01
-77_-76ins12; *-73T>G; -42T>C; *-27T>C	1	1	0.01
-77_-76ins12; -42T>C	1	1	0.01
-77_-76ins24; *-76G>T; -72_-67del6; -42T>C; *-27T>C	1	1	0.01
-77_-76ins24; -72_-67del6; -42T>C; -37_-27del11	1	1	0.01
*-73T>G; -37_-27del11	1	1	0.01
*-91G>T; *-27T>C	1	1	0.01
**Total (17 haplotypes)**			1

*novel variants

**ʵ** the number of haplotypes differ from the number of dogs because some individuals were heterozygous while others were homozygous

ʞ Frequency = (number of haplotypes/136 dog chromosomes)

Breeds that were reported to have had 5’UTR *Cox-2* variants concurrent with RD [4], were represented in this cohort by the Boxer (n = 4), with variants *Cox2–*72_-67del6, *Cox2* -42T>C and *Cox2–*37_-27del11; Bernese Mountain dog (n = 2) *Cox2–*77_-76ins12, *Cox2–*72_-67del6, *Cox2* -42T>C and *Cox2–*37_-27del11; Australian Shepherd (n = 1) *Cox2–*77_-76ins12; Labrador Retriever (n = 2) *Cox2–*77_-76ins30 and *Cox2*–72_-67del6, *Cox2* -42T>C and *Cox2–*37_-27del11; Yorkshire Terrier (n = 1) *Cox2–*77_-76ins30; Rottweiler (n = 1) *Cox2–*77_-76ins24; and German Shepherd dogs (n = 10) *Cox2–*72_-67del6, *Cox2* -42T>C and *Cox2–*37_-27del11(n = 3). We sequenced unaffected Shetland Sheepdog (n = 9) and found that all of the individuals tested had regional variants, and dogs from this breed had haplotypes that recombine variants *Cox2–*77_-76ins30/24/12 and *Cox2–*72_-67del6, *Cox2* -42T>C and *Cox2–*37_-27del11 (S1 Table).

The FCR breed was represented by the largest cohort (n = 84). The sequence variants identified within FCRs are listed in Table 2 by nucleotide position and haplotype frequency. The most frequent haplotype documented within the FCRs was CanFam3.1 assembly sequence at allele frequency of 0.50, followed by a novel haplotype found in FCRs, *Cox*2-72_-67del6; -42T>C; -27T>C, at a frequency of 0.38. Less frequent were a second novel haplotype unique to FCRs, Cox2-77_-76ins24; -67_-66dup6 (0.06), and Cox2-72_-67del6; -42 T>C; -37_-27del11 (0.06). The novel haplotypes found in FCRs brought the total number of haplotypes observed in dogs to 19.

**Table 2:** 5’UTR *Cox-2* variants found in 84 Flat-Coated Retrievers.

Haplotype	# of dogs	# of haplotypesʵ	Frequencyʞ
CanFam3.1 assembly sequence	62	84	0.50
-72_-67del6; -42 T>C; *-27T>C	51	64	0.38
-77_-76ins24; *-67_-66dup6	10	10	0.06
-72_-67del6; -42 T>C; -37_-27del11	10	10	0.06
**Total (4 haplotypes)**			1

*novel variants

**ʵ** the number of haplotypes differ from the number of dogs because some individuals were heterozygous while others were homozygous

ʞ Frequency = (number of haplotypes/168 FCR chromosomes)

In order to clarify the ancestral state of the 5’ UTR sequence of canine *Cox-2*, 12 DNA samples from gray wolves were sequenced. Based on STRs, a matrix of maximum likelihood estimates of relatedness revealed that 7 of the 12 wolves were unrelated. The 5’UTR haplotypes of the 7 unrelated wolves are presented in Table 3. The most common haplotype observed within wolves was sequence variant *Cox2–*77_-76ins12 at allele frequency of 0.72. A single wolf was heterozygous for a novel variant *Cox2–*77_-76ins19, and two novel SNPs were identified in wolves; *Cox2* -67G>T, and *Cox2* -62T>G (Table 3). A single wolf was heterozygous for CanFam3.1 assembly sequence. The five remaining wolves shared ancestry and all of them were homozygous for *Cox2–*77_-76ins12. The wolves sampled came from a genetically diverse population based on high heterozygosity values observed for the STR markers (S4 Table).

**Table 3:** 5’UTR *Cox-2* variants found in 7 Alaskan gray wolves.

Haplotype	# of Wolves	#of haplotypesʵ	Frequencyʞ
-77_-76ins12	7	11	0.79
*-77_-76ins19	1	1	0.07
*-67G>T; *-62T>G	1	1	0.07
CanFam3.1 assembly sequence	1	1	0.07
**Total (4 haplotypes)**			1

*novel variants

**ʵ** the number of haplotypes differ from the number of dogs because some individuals were heterozygous while others were homozygous

ʞ Frequency = (number of haplotypes/14 wolf chromosomes)

**Fig 2:**
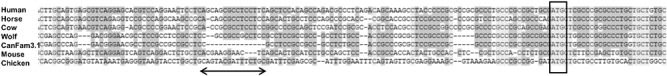
Alignment of DNA sequences upstream of the ATG of the *Cox-2* gene from Human, Horse, Cow, Wolf, CanFam3.1 assembly, Mouse and Chicken. The presumed translation initiation ATG is boxed. A DNA variant (deletion of 12 nucleotides) is observed in CanFam3.1 sequence when compared to other organisms (double headed arrow).

We next compared *Cox-2* 5’UTR sequences across species. mRNA sequences from Human (Homo sapiens; accession: U04636), Horse (Equus caballus; AF027334), Cow (Bos taurus; DT845401), Mouse (Mus musculus; BB653429), and Chicken (Gallus gallus; NM_001167719) were aligned together with genomic sequences from Wolf (Canis lupus; KP296709), and CanFam3.1 sequence (Canis lupus familiaris; canFam3_dna range = chr7:19674506–19674874). The alignment, shown in Fig 2, demonstrates that a conserved regional sequence includes ~12 additional nucleotides when compared to the CanFam3.1 assembly.

## Discussion

The genomic region immediately upstream of the *Cox-2* gene was sequenced in 3 groups of dogs free of clinical signs of renal disease as well as in gray wolves. The first group of dogs consisted of unaffected individuals cleared by necropsy. To increase the sample size we included non-azotemic dogs older than 6 years of age because RD is usually documented in juvenile animals [16] [23]. However, there are reports of older cases [8], and age of onset could be a breed specific trait. The third group of dogs consisted of FCRs free of clinical signs according to owner questionnaire without medical records. While a limitation of the study is the incomplete phenotyping of some of the dogs, the results were similar between the groups.

Approximately half of the haplotypes observed in dogs represented CanFam3.1 assembly, while the other half featured a high degree of polymorphism including insertions, duplications, deletions and SNPs. Novel variants were a common observation and a total of 11 new variants were described. Surprisingly, the wolves sampled showed lower levels of regional polymorphism when compared to dogs and 11 of the 12 wolves had sequence variant *Cox2*–77_-76ins12. While the number of wolf samples available for this study is insufficient to draw final conclusions, the presence of fewer wolf haplotypes than dogs’ was an unexpected result. This is because the wolves showed high levels of genetic diversity at 24 genome-wide STR markers and because the wolf genome is more polymorphic than the dog’s [26]. Additional sequences from wolves may elucidate this observation.

Across species alignment of the regional DNA sequence revealed that a haplotype with additional 12 bases, comparable to -77_-76ins12, is conserved between gray wolves, Human, Horse, Cow, Mouse and Chicken. This suggests that this is the wildtype haplotype, particularly since this was also the second most common haplotype found in non-azotemic dogs. The wild-type sequence is not expected to increase the likelihood of RD, challenging the claim that this variant and/or other regional sequence variants harbor susceptibility to RD in dogs. Based on our results, we propose that additional studies are needed in order to uncover RD susceptibility loci to assist dog breeders in selecting against RD in predisposed breeds.

Currently, the functional significance of the regional sequence variants is unknown. The canine *Cox-2* promoter has been identified and employed successfully by Rachakonda et al (2008) and Rai et al (2011) in in-vitro arthritis models using canine chondrocytes [34, 35]. Only a short region of ~64 bases at the 3' end of this canine *Cox-2* promoter overlaps with the region containing the variants reported by Whiteley et al (2011) [4]. Although the overlapping region contains sequences that may correspond to SP1 sites (CpG islands), Nunez et al (2011) have identified the main regulatory regions to be further upstream in the human *Cox-2* gene [36]. *Cox-2* basal level expression is variable among individual dogs [37], but variability in expression is also noted within a species with fewer documented 5’UTR sequence polymorphism such as people [38, 39].

An explanation for the presence of multiple DNA variants immediately upstream to the presumed translation initiation site of canine *Cox-2* in dogs requires further investigation. Sequence polymorphisms within the 5’UTR of other canine genes have been studied and show moderate variation comparing to the various haplotypes observed within the canine *Cox-2* gene. Young and Bannasch (2008) acquired 5’UTR sequences from 24 unrelated dogs and revealed a single SNP each in the *SHOX* and *RMRP* genes [40], and Shirazi-Beechey et al (2013) screened 60 dogs to reveal 3 SNPs within the 5’UTR region of the *SGLT1* gene [41]. It is possible that this genomic region represents a recombination hotspot, as Auton et al (2013) studied recombination hotspots in dogs and observed elevated rates of recombination around transcription start sites. It has been reported that CpG content is strongly associated with canine recombination and that the most recombinogenic repeats are low-complexity with high levels of GC and CpG content [42], as found in this region.

## Ethic Statement

Samples donated to the AHT with owner consent for use in variable research projects from Flatcoated Retrievers were submitted in the form of buccal swabs or blood. Buccal swabs were collected by owners using a non-invasive method. Blood samples were the remnants of blood drawn by veterinarians for routine and/or diagnostic veterinary purposes, and were not collected specifically for the purposes of research.

For the portion of the study carried out in the University of California, the sample collection protocol was approved by the University of California, Davis Animal Care and Use Committee (protocol #16892), with owner consent. Additional samples came from blood samples that were drawn for medical purposes at the Veterinary Teaching Hospital, UC Davis. DNA was extracted from unused portions of the samples. Tissue samples from dogs that underwent complete necropsy were used for research purposes with owner’s consent.

Gray wolves (Canis lupus) samples collected from the Yukon Territory, Canada, were archived for over 10 years and used previously [28]. No samples were collected specifically for the purpose of this study and no animals were killed specifically for this study.

## Supporting Information

S1 Table Breed representation of the 19 haplotypes found within the 5’UTR of Cox-2 in 152 dogs. *novel variants.
(DOCX)

S2 Table 5’UTR *Cox-2* variants found in 46 dogs that had normal blood urea nitrogen and creatinine levels and no history of renal disease. *novel variants.
(DOCX)

S3 Table 5’UTR *Cox-2* variants found in 22 dogs that underwent complete necropsy and were clear of RD. *novel variants.
(DOC)

S4 Table Heterozygosity values observed in 12 Alaskan gray wolves using the canine parental verification panel at the *VGL. PIC: Polymorphism Information Content (a value derived to measure the infomativeness of a genetic marker). * https://www.vgl.ucdavis.edu/.
(DOCX)
