# Correction: DNA Sequence Variants in the Five Prime Untranslated Region of the Cyclooxygenase-2 Gene Are Commonly Found in Healthy Dogs and Gray Wolves

**DOI:** 10.1371/journal.pone.0195924

**Published:** 2018-04-10

**Authors:** Noa Safra, Louisa J. Hayward, Miriam Aguilar, Benjamin N. Sacks, Jodi L. Westropp, F. Charles Mohr, Cathryn S. Mellersh, Danika L. Bannasch

The authors acknowledge that variant -77_-76ins30, identified independently in DNA sequences in this study (1), was also published in a 2001 patent (2) and posted in Genbank under Accession AX082874.

In addition, the authors note that a related study was not discussed or cited in the published *PLOS ONE* article. In an article titled “Allelic variation in the canine Cox-2 promoter causes hypermethylation of the canine Cox-2 promoter in clinical cases of renal dysplasia” (3), the author used DNA from 13 dogs to query methylation within short DNA sequences in the 5’UTR region of the Cox-2 gene. The study uncovered methylated sequence variants upstream to the Cox-2 gene, in a few clinical cases of canine renal dysplasia. However, unmethylated DNA was found regardless of sequence variations. The biological significance of these short DNA sequences was previously discussed in an article for which post-publication concerns were raised regarding the reliability of the findings (4, 5).

Compared to these previous studies (3–5), we investigated the same region on CanFam3.1 CFA7: 19,664,240–19,664,662, and observed 11 DNA sequence variations in addition to the variants tested previously for methylation status (1). The marked sequence diversity totaled 17 different haplotypes in 68 dogs, and no statistically significant differences were noted between the different phenotypes. Our interpretation is that there is no relation between different sequence variations in the 5’UTR of canine Cox-2 and canine renal dysplasia. The random methylation pattern in (3) provides yet another piece of evidence to support a benign role for these DNA variants.

In response to questions raised after publication, the authors would also like to clarify the method used in aligning sequences in our study. Different sequence alignments can be easily explained when the biological function of the sequence is considered. The regional DNA sequence is GC-rich typical of 5’UTR region, with multiple repeats and indel sequence polymorphisms. These qualities present computational challenges making an algorithm-based alignment less reliable (6). Therefore, we aligned the sequences based on the ‘ATG’ start codon. This method allows the alignment to be anchored by a known highly conserved sequence. The verification for ‘correct’ alignment is seen to the right of the ATG where many transcripts including Cox-2, are highly conserved across species.
