# Supplementary material for: DNA Sequence Variants in the Five Prime Untranslated Region of the Cyclooxygenase-2 Gene Are Commonly Found in Healthy Dogs and Gray Wolves
Source: PLoS One. 2015 Aug 5;10(8):e0133127. doi: 10.1371/journal.pone.0133127 (PMC4526539; doi:10.1371/journal.pone.0133127)
Supplement: S1 Table — *novel variants. (DOCX) [file pone.0133127.s001.docx]

Table S1: Breed representation of the 19 haplotypes found within the 5’UTR of *Cox-2* in 152 dogs.

| **Breed** | **#of dogs** | **#of haplotypesʵ** | **Haplotype** |
| --- | --- | --- | --- |
| Alaskan Malamute | 1 | 1 | CanFam3.1 sequence |
| Australian Cattle Dog | 1 | 1 |  |
| Beagle | 1 | 1 |  |
| Bernese Mountain Dog | 1 | 1 |  |
| Bichon Frise | 2 | 2 |  |
| Boxer | 4 | 5 |  |
| Brittany Spaniel | 1 | 1 |  |
| Doberman Pinscher | 1 | 1 |  |
| English Bulldog | 1 | 2 |  |
| English Springer Spaniel | 1 | 1 |  |
| Flat-Coated Retriever | 62 | 84 |  |
| German Shepherd Dog | 10 | 16 |  |
| Golden Retriever | 1 | 1 |  |
| Labrador Retriever | 1 | 1 |  |
| Miniature Pinscher | 1 | 2 |  |
| Mixed Breed | 5 | 5 |  |
| Pug | 2 | 2 |  |
| Rat Terrier | 1 | 1 |  |
| Scottish Terrier | 1 | 1 |  |
| Shetland Sheepdog | 5 | 6 |  |
| Toy Fox Terrier | 1 | 2 |  |
| Soft Coated Wheaten Terrier | 1 | 1 |  |
| Weimaraner | 1 | 1 |  |
| Australian Shepherd | 1 | 2 | -77_-76ins12 |
| Bernese Mountain Dog | 1 | 1 |  |
| Bichon Frise | 2 | 2 |  |
| Border collie | 1 | 2 |  |
| Brittany Spaniel | 1 | 1 |  |
| Cocker Spaniel | 1 | 2 |  |
| Dachshund | 1 | 2 |  |
| Doberman Pinscher | 2 | 3 |  |
| English Springer Spaniel | 1 | 1 |  |
| Golden Retriever | 1 | 1 |  |
| Miniature Pinscher | 1 | 2 |  |
| Mixed Breed | 3 | 4 |  |
| Pug | 1 | 1 |  |
| Scottish Terrier | 1 | 1 |  |
| Shetland Sheepdog | 6 | 9 |  |
| Siberian Husky | 1 | 2 |  |
| Soft Coated Wheaten Terrier | 1 | 1 |  |
| Weimaraner | 1 | 1 |  |
| Bernese Mountain Dog | 1 | 2 | -72_-67del6; -42T>C; -37_-27del11 |
| Boxer | 1 | 1 |  |
| Chihuahua | 1 | 2 |  |
| Flat-Coated Retriever | 10 | 10 |  |
| German Shepherd Dog | 3 | 3 |  |
| Labrador Retriever | 1 | 2 |  |
| Pit Bull Terrier | 1 | 2 |  |
| Bull Mastiff | 1 | 2 | -77_-76ins24 |
| Mixed Breed | 1 | 1 |  |
| Pug | 1 | 1 |  |
| Rottweiler | 1 | 2 |  |
| Labrador Retriever | 1 | 1 | *-77_-76ins30 |
| Mixed Breed | 1 | 2 |  |
| Shetland Sheepdog | 1 | 1 |  |
| Yorkshire Terrier | 1 | 2 |  |
| Boxer | 2 | 2 | -37_-27del11 |
| Rat Terrier | 1 | 1 |  |
| Mixed Breed | 1 | 2 | -77_-76ins12; -77_-76ins24 |
| Mixed Breed | 1 | 2 | -72_-67del6; -42T>C; *-37_-27dup11 |
| Mixed Breed | 1 | 1 | -77_-76ins12; -42T>C; -37_-27del11 |
| Alaskan Malamute | 1 | 1 | *-67G>T; -42T>C; -37_-27del11 |
| Shetland Sheepdog | 1 | 1 | -77_-76ins12; -72_-67del6; -37_-27del11 |
| Golden Retriever | 1 | 1 | -77_-76ins12; *-73T>G;-42T>C; *-27T>C |
| Flat-Coated Retriever | 51 | 64 | -72_-67del6; -42 T>C; *-27T>C |
| Flat-Coated Retriever | 10 | 10 | -77_-76ins24 ; *-67_-66dup6 |
| Mixed Breed | 1 | 1 | -77_-76ins12; -42T>C |
| Beagle | 1 | 1 | -77_-76ins24; *-76G>T; -72_-67del6; -42T>C; *-27T>C |
| Shetland Sheepdog | 1 | 2 | -77_-76ins24; -72_-67del6; -42T>C; -37_-27del11 |
| Mixed Breed | 1 | 1 | *-73T>G; -37_-27del11 |
| German Shepherd Dog | 1 | 1 | *-91G>T; *-27T>C |

* Novel variants **ʵ** the number of haplotypes differ from the number of dogs because some individuals were heterozygous while others were homozygous
