# Supplementary material for: DNA Sequence Variants in the Five Prime Untranslated Region of the Cyclooxygenase-2 Gene Are Commonly Found in Healthy Dogs and Gray Wolves
Source: PLoS One. 2015 Aug 5;10(8):e0133127. doi: 10.1371/journal.pone.0133127 (PMC4526539; doi:10.1371/journal.pone.0133127)
Supplement: S2 Table — *novel variants. (DOCX) [file pone.0133127.s002.docx]

**Table S2:** 5’UTR *Cox-2* variants found in 46 dogs that had normal blood urea nitrogen and creatinine levels and no history of renal disease

| **Haplotype** | **# of dogs** | **# of haplotypes** | **Frequency** |
| --- | --- | --- | --- |
| CanFam3.1 assembly sequence | 31 | 37 | 0.40 |
| -77_-76ins12 | 19 | 26 | 0.28 |
| -72_-67del6 ; -37_-27del11 ; -42T>C | 7 | 10 | 0.11 |
| -77_-76ins24 | 3 | 5 | 0.05 |
| *-77_-76ins30 | 2 | 3 | 0.03 |
| -77_-76ins12; -77_-76ins24 | 1 | 2 | 0.02 |
| -72_-67del6; -42T>C; *-37_-27dup11 | 1 | 2 | 0.02 |
| -77_-76ins12; -72_-67del6; -37_-27del11 | 1 | 1 | 0.01 |
| -77_-76ins24 ; *-76G>T ; -72_-67del6 ; -42T>C ; *-27T>C | 1 | 1 | 0.01 |
| -77_-76ins24 ; -72_-67del6 ; -42T>C ; -37_-27del11 | 1 | 1 | 0.01 |
| *-91G>T ; *-27T>C | 1 | 1 | 0.01 |
| **Total (11 haplotypes)** | | | 1 |

*novel variants
