# Supplementary material for: DNA Sequence Variants in the Five Prime Untranslated Region of the Cyclooxygenase-2 Gene Are Commonly Found in Healthy Dogs and Gray Wolves
Source: PLoS One. 2015 Aug 5;10(8):e0133127. doi: 10.1371/journal.pone.0133127 (PMC4526539; doi:10.1371/journal.pone.0133127)
Supplement: S3 Table — *novel variants. (DOC) [file pone.0133127.s003.doc]

**Table S3:** 5’UTR *Cox-2* variants found in 22 dogs that underwent complete necropsy and were clear of RD*

| **Haplotype** | **# of dogs** | **# of haplotypes** | **Frequency** |
| --- | --- | --- | --- |
| CanFam3.1 assembly sequence | 13 | 16 | 0.36 |
| -77_-76ins12 | 8 | 11 | 0.25 |
| -72_-67del6; -37_-27del11; -42T>C | 1 | 2 | 0.05 |
| *-67G>T; -42T>C; -37_-27del11 | 1 | 2 | 0.05 |
| -77_-76ins24 | 1 | 1 | 0.02 |
| *-77_-76ins30 | 2 | 3 | 0.07 |
| -37_-27del11 | 3 | 5 | 0.11 |
| -77_-76ins12 ; *-73T>G ; -42T>C ; *-27T>C | 1 | 1 | 0.02 |
| -77_-76ins12 ; -42T>C ; -37_-27del11 | 1 | 1 | 0.02 |
| -77_-76ins12; -42T>C | 1 | 1 | 0.02 |
| *-73T>G ; -37_-27del11 | 1 | 1 | 0.02 |
| **Total (11 haplotypes)** | | | 1 |

*novel variants
