# Supplementary material for: DNA Sequence Variants in the Five Prime Untranslated Region of the Cyclooxygenase-2 Gene Are Commonly Found in Healthy Dogs and Gray Wolves
Source: PLoS One. 2015 Aug 5;10(8):e0133127. doi: 10.1371/journal.pone.0133127 (PMC4526539; doi:10.1371/journal.pone.0133127)
Supplement: S4 Table — PIC: Polymorphism Information Content (a value derived to measure the infomativeness of a genetic marker). * https://www.vgl.ucdavis.edu/. (DOCX) [file pone.0133127.s004.docx]

**Table S4:** Heterozygosity values observed in 12 Alaskan gray wolves using the canine parental verification panel at the *VGL. PIC: Polymorphism Information Content (a value derived to measure the infomativeness of a genetic marker).

| **STR marker** | **Number of alleles** | **PIC value** | **Expected heterozygosity** | **Observed heterozygosity** |
| --- | --- | --- | --- | --- |
| AHT121 | 8 | 0.79 | 0.86 | 0.92 |
| AHT137 | 8 | 0.76 | 0.82 | 0.75 |
| AHTH130 | 4 | 0.65 | 0.74 | 0.50 |
| AHTh171-A | 5 | 0.68 | 0.76 | 0.75 |
| AHTh260 | 7 | 0.79 | 0.86 | 0.82 |
| AHTk211w | 4 | 0.59 | 0.66 | 0.58 |
| AHTk253 | 4 | 0.62 | 0.71 | 0.58 |
| C22.279 | 4 | 0.51 | 0.61 | 0.67 |
| FH2001 | 4 | 0.61 | 0.71 | 1.00 |
| FH2054 | 9 | 0.84 | 0.89 | 0.92 |
| FH2328 | 6 | 0.70 | 0.76 | 0.83 |
| FH2848 | 5 | 0.69 | 0.76 | 0.75 |
| INRA21 | 6 | 0.78 | 0.84 | 0.58 |
| INU005 | 4 | 0.68 | 0.76 | 0.92 |
| INU030 | 5 | 0.69 | 0.76 | 0.75 |
| INU055 | 5 | 0.72 | 0.80 | 1.00 |
| LEI004 | 5 | 0.68 | 0.76 | 1.00 |
| REN105L03 | 6 | 0.74 | 0.81 | 0.83 |
| REN162C04 | 8 | 0.80 | 0.86 | 0.92 |
| REN169D01 | 5 | 0.59 | 0.66 | 0.73 |
| REN169O18 | 6 | 0.76 | 0.83 | 0.92 |
| REN247M23 | 5 | 0.64 | 0.72 | 0.67 |
| REN54P11 | 4 | 0.51 | 0.62 | 0.25 |
| REN64E19 | 7 | 0.66 | 0.72 | 0.83 |

* https://www.vgl.ucdavis.edu/
